# Modeling Trust in COVID-19 Contact-Tracing Apps Using the Human-Computer Trust Scale: Online Survey Study

**DOI:** 10.2196/33951

**Published:** 2022-06-13

**Authors:** Sonia Sousa, Tiina Kalju

**Affiliations:** 1 School of Digital Technologies Tallinn University Tallinn Estonia; 2 Institute for Systems and Computer Engineering, Technology and Science University of Trás-os-Montes and Alto Douro Vila Real Portugal

**Keywords:** human-computer interaction, COVID-19, human factors, trustworthy AI, contact-tracing, app, safety, trust, artificial intelligence, Estonia, case study, monitoring, surveillance, perspective, awareness, design, covid, mobile app, mHealth, mobile health

## Abstract

**Background:**

The COVID-19 pandemic has caused changes in technology use worldwide, both socially and economically. This pandemic crisis has brought additional measures such as contact-tracing apps (CTAs) to help fight against spread of the virus. Unfortunately, the low adoption rate of these apps affected their success. There could be many reasons for the low adoption, including concerns of security and privacy, along with reported issues of trust in CTAs. Some concerns are related with how CTAs could be used as surveillance tools or their potential threats to privacy as they involve health data. For example, in Estonia, the CTA named HOIA had approximately 250,000 downloads in the middle of January 2021. However, in 2021, only 4.7% of the population used HOIA as a COVID-19 CTA. The reasons for the low adoption include lack of competency, and privacy and security concerns. This lower adoption and the lack of trustworthiness persist despite efforts of the European Union in building ethics and trustworthy artificial intelligence (AI)-based apps.

**Objective:**

The aim of this study was to understand how to measure trust in health technologies. Specifically, we assessed the usefulness of the Human-Computer Trust Scale (HCTS) to measure Estonians’ trust in the HOIA app and the causes for this lack of trust.

**Methods:**

The main research question was: Can the HCTS be used to assess citizens’ perception of trust in health technologies? We established four hypotheses that were tested with a survey. We used a convenience sample for data collection, including sharing the questionnaire on social network sites and using the snowball method to reach all potential HOIA users in the Estonian population.

**Results:**

Among the 78 respondents, 61 had downloaded the HOIA app with data on usage patterns. However, 20 of those who downloaded the app admitted that it was never opened despite most claiming to regularly use mobile apps. The main reasons included not understanding how it works, and privacy and security concerns. Significant correlations were found between participants’ trust in CTAs in general and their perceived trust in the HOIA app regarding three attributes: competency (*P*<.001), risk perception (*P*<.001), and reciprocity (*P*=.01).

**Conclusions:**

This study shows that trust in the HOIA app among Estonian residents did affect their predisposition to use the app. Participants did not generally believe that HOIA could help to control the spread of the virus. The result of this work is limited to HOIA and health apps that use similar contact-tracing methods. However, the findings can contribute to gaining a broader understanding and awareness of the need for designing trustworthy technologies. Moreover, this work can help to provide design recommendations that ensure trustworthiness in CTAs, and the ability of AI to use highly sensitive data and serve society.

## Introduction

### Background

The COVID-19 pandemic has changed how we view technology as a resource to stop the spread of disease. To address the need to control the spread of the virus, many governments and public health authorities worldwide have launched several technological initiatives, including the development of artificial intelligence (AI) contact-tracing mobile apps (CTAs). As a result, by the end of 2020, there were more than 50 CTAs available in both Google Play and iOS App Store [1,2]. According to Nguyen et al [3], security and privacy are crucial in designing AI-based CTA technologies. If users perceive CTAs as a threat to their privacy, this might affect their predisposition to use the app, ultimately affecting its adoption rate and tool effectiveness. This evidence has led to an increased discourse for design systems toward focusing on ensuring that CTAs are secure and private. Previous studies have recommended several criteria such as ensuring a low level of complexity of the security feature so that it is easy to use and understandable for the general population [4,5], visibility and interaction from the user, and unambiguous and clear messages to follow while designing security measures [6-8]. Similar arguments were put forth in Europe’s stated goals to ensure ethical and responsible technological development. Although COVID-19 CTAs in Europe followed the General Data Protection Regulation and ISO/IEC 27001 [9] regulations, and were also designed in consideration of current AI principles to regulate technology use (ie, Ethical guidelines for Trustworthy AI [10]), this was not sufficient to ensure the trustworthiness from citizens. This lack of trustworthiness exists despite widely available information on how these technologies were built with transparent and ethical principles in mind. Moreover, despite government initiatives to push through their adoption, the download rates and actual usage rates of these apps remained low [2,6,11-13]. One reason for this low adoption might be that security and privacy in computer science are still mainly approached from a technical perspective [14]. Privacy attributes in technology can be more profound and complex than technical qualities. Privacy is defined as a person’s control over the information that is manipulated and communicated to others [6,15-18].

Privacy also includes interpersonal characteristics such as the perception of privacy, system honesty or benevolence communication, and shared control to minimize associated risk and uncertainty. For instance, despite appropriate regulations and principles being considered when designing Estonia’s COVID-19 CTA (HOIA), the adoption of HOIA by citizens did not increase. The critical reasons for the low adoption of HOIA included lack of effectiveness (10%) and concerns of security and privacy (19%) according to a survey initiated by The Ministry of Social Affairs, surveying 92% of Estonian residents [13,19]. Thus, all efforts made in designing AI-based transparent and ethically responsible CTAs that can prevent data misuse and ensure the development of responsible trustworthy AI interactions were unsuccessful.

We believe that it is essential to find new ways to ensure incorporating trust values in the design of such apps that could lead to building more technological, socially responsible societies. One should expect trust to be increasingly in demand as a means of enduring the complexity of a future that technology will generate. The quality and depth of technology use are also significantly affected by users’ trust in the technology. Trust is defined according to the ability to determine who to trust, and represents the willingness of a party to be vulnerable to the actions of another party based on the expectation that the other will perform a particular action important to the trustor, irrespective of the ability to monitor or control that other party [20-22].

### Research Gaps

Prior research confirms that technology acceptance and adoption are affected by the level of trust users have in the technology [11,20-23]. However, evidence shows that designing trustworthy technologies is complex and needs to be better understood. Like privacy, trust is an interpersonal quality that is present in many moments of our daily lives, and is thus often considered unconsciously. Whether being conscious or unconscious of its existence, trust represents an important key of the relationships encountered in daily life, including interactions between humans and machines. Establishing a trustful relationship implies peoples’ permission to share knowledge, delegation, and cooperative actions [11,22,24,25]. Thus, in addition to the current research challenge for ensuring that all ethical, privacy, and technical security requirements are considered [5,7,9], we argue that trust might be the reason why users do not feel comfortable using CTAs that depend on citizens’ data to function properly. If this is indeed the case, besides existing design regulations and principles, designers will also need mechanisms to analyze individuals’ perceived trustworthiness in AI apps. In this way, designers and other stakeholders can gain a deeper understanding of how individuals perceive the benefits of AI, and assess their predisposition to cooperate and be more willing to use the technologies. Thus, it is important to gauge the extent to which such AI data–driven technologies are perceived as trustworthy (ie, the gains of using CTAs are higher than the possible losses).

There are three main rationales for the above argument. First, with the current culture of increased introduction and use of complex systems in our daily activities, researchers need to focus more on conceiving responsible human-computer interactions. Second, current paradigms supporting ethical and responsible design practices are insufficient to ensure technology trustworthiness. Third, a new human-machine interaction mechanism is needed to effectively evaluate users’ trust perceptions in technology (eg, assess users’ experience toward incorporated trust values). Namely, we propose new human-centered design frameworks and mechanisms to guide the design and technology evaluation process. Overall, in the past decade, human-computer interaction has contributed significantly toward improving the quality of living with technology. Consequently, regular individuals are getting more involved, engaged, and dependent on technology to achieve their goals. It is true that we no longer live without technology. Despite this, the above arguments indicate that we are entering a new era that depends on data to thrive. This symbiotic dependence of humans in systems abilities and of systems dependence in our data to provide meaningful information has increased the complexity of the technology provided. Consequently, we have become more reliant on trust to survive in these complex symbiotic relationships. This is clearly shown in how digital CTAs were affected by these symbiotic relationships. Most of these apps are collecting highly sensitive data from individuals, including where they have been and with whom they have been in contact.

## Methods

### Study Aims and Design

This study builds on the prior work of Gulati et al [20] and Sousa et al [22], and is guided by one central research question: Can the Human-Computer Trust Scale (HCTS) be used to assess an individual’s perception of trust in health technologies? The main goal of this study was to propose a novel design evaluation mechanism to incorporate trust values in health care technologies, and make health care interventions and technologies more trustworthy and accepted. Namely, we used partial least-squares structural equation modeling (PLS-SEM) to empirically ascertain which attributes of the proposed scale (HCTS) hold in health care contexts and can be used as lenses to evaluate individuals’ trust predisposition to interact. The study was divided into two main stages: (1) adaptation and translation of the scale, and (2) measurement and validation of the questionnaire (HCTS).

### Theoretical Model

**Figure 1:**
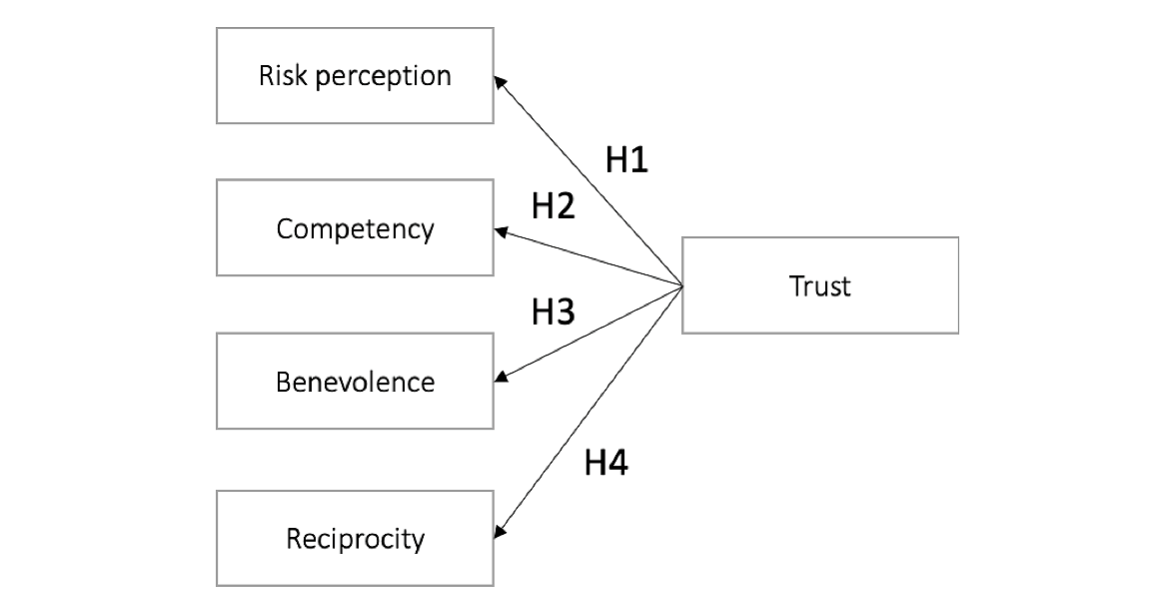
Human-computer trust model under investigation. H: Hypothesis.

The adopted theoretical model, the HCTS [20], illustrates the multidimensional nature of trust, taking into account several attributes of trust, as shown in Figure 1. This model was validated with statistical modeling techniques. The proposed attributes of the model were gathered from a systematic multidisciplinary literature review, combined with (1) a word elicitation study to capture a rich set of multidisciplinary notions encapsulating trust; (2) participatory design sessions and exploratory interviews with users to further identify antecedents of trust; (3) the unification of technology acceptance models [22]; and (4) separate studies to ensure statistical certainty of the scale proposed: trust in Siri, trust in the Estonian electronic voting system, trust in futuristic scenarios, and trust in human-robot interaction [20,26]. The final scale to measure trust consists of three main attributes: risk perception, competency, and benevolence. In line with the above findings and with the awareness that trust assessment is context- and culture-dependent, we assessed the validity of the scale to measure citizens’ trust attitudes in CTAs. To achieve our goal, we developed four sets of assumptions that might affect or predict a user’s trust when interacting with the HOIA app. The four hypotheses (H1-H4) established in regard to our main research question are outlined in Textbox 1.

**Hypotheses of the study.**

**Hypothesis 1**

- There is a significant and positive association between risk perception in the HOIA app and general trust in HOIA. Risk perception is defined as *the extent to which one party is willing to participate in a given action while considering the risk and incentives involved*. Here, we assumed that the extent to which individuals are willing to participate in a given action (ie, to use HOIA) while considering that the risk and incentives involved are directly associated with their perception of technology trustworthiness: with a higher perceived risk, there will be less willingness to interact; with a lower perceived risk, users will be more willing to interact.

**Hypothesis 2**

- There is a significant and positive association between competence and general trust in HOIA. HOIA competence is defined as *the ease of use associated with the use of a system in that it is perceived to perform its tasks accurately and correctly*. Here, we assumed that an individual’s perception of a contact-tracing app as competent is based on its functionality, closely linked to the concept of usefulness of a system. Higher perceived competency indicates that participants perceived the tool to be capable of doing what is expected, be useful, and will help them achieve desired goals.

**Hypothesis 3**

- There is a significant and positive association between benevolence and general trust in HOIA. Benevolence is defined as a citizen’s *perception that a particular system will act in their best interest and that most people using the system share similar social behaviors and values*. Here, we assumed that an individual’s perception that a particular system will act in their best interest, and that most people using the system share similar social behaviors and values that a particular technology will provide. Higher perceptions of benevolence are associated with fewer risks and uncertainties in its use.

**Hypothesis 4**

- There is a significant and positive association between reciprocity and trust in HOIA use. The notion of reciprocity is understood as *the degree to which an individual sees oneself as a part of a group*. It is built on the principle of mutual benefit, feeling a sense of belonging, and feeling connected, based on the give-and-take principles associated with the notion of computers as social actors. Here, we assumed that a citizen’s perception of contact tracing apps is reciprocal based on the degree to which an individual sees oneself as a part of a group.

### Study Procedure

#### Questionnaire

We used a semistructured questionnaire to collect data. Before distributing the questionnaire, we adapted the original scale to the context and translated the content from English into Estonian. The translation and adaptation of the instrument followed the guidelines of the adaptation, translation, and validation process [27]. The survey was designed based on the HCTS in the Estonian language and was administered during April 2021. The objective of this study was to build on prior works and empirically assess HCTS to ascertain which attributes of the model hold true in health user–technology interactions.

The survey was created using both Lime Survey and Google Forms. During the pilot study, the feedback from the respondents was that the visual design of the Google Forms is less confusing; therefore, it was decided to adopt Google Forms as the final survey format.

#### Stimuli

To ensure that all participants understood the technical artefact in question and their perceptions of trust regarding similar experiences, we provided the official video that explains HOIA to the users as a stimulus, following the concept of technology probe and design fiction, also known as a vignette-based study in psychology.

#### Recruitment

The survey was carried out among the Estonian population, which was distributed online, mainly through Facebook and other social network groups available to the authors. A convenience sample was used in data collection because this enables reaching members of the population who are easily accessible, available, and willing to participate [28].

### Ethical Considerations

This study complies with the basic ethical principles for the responsible conduct of research involving human subjects. Informed consent was requested from all participants, and authorization was obtained from the authors of the scale [20] to carry out the contextual adaptation and validation of the scale. The study was approved by the Tallinn University Ethics committee on July 9th, 2021 (study name: “Survey on the dynamic trust relationships between technology, society and culture"; approval number: Taotlus nr 6-5.1/17).

## Results

### Participant Characteristics

A total of 78 responses were obtained and used for data analyses; very few responses were excluded as all respondents fully completed the survey. The three excluded cases included answers leaning in majority toward neutral options. Data collected included the following information: demographics, usage patterns of mobile apps and HOIA, trust in HOIA (including risk perception, benevolence, competence, and general trust), and opinions about HOIA’s existing and additional functionalities. Among the 78 respondents, 73% (n=57) were women and only 27% (n=21) were men. Almost half of the respondents (36/78, 47%) were between the ages of 31-42 years and approximately one third (25/78, 32%) were 43-55 years old.

### HOIA Usage Patterns

Among the 78 respondents, 61 had downloaded the HOIA CTA. Among them, the 47 women showed the highest rate of downloads compared with the 14 male respondents. Younger respondents (aged 18-30 years) had a higher number of downloads (88%), but they also represented the smallest sample. Slightly more than half of the participants (56%) admitted that they do not feel confident in how to use HOIA; this perception was more prominent among men (n=13). Twenty participants admitted that they had never opened the app, despite 61 claiming to use mobile apps daily.

Among the 17 respondents who had not downloaded the HOIA app, the majority were men. The main reasons claimed by participants for not downloading HOIA included: do not understand how it works, and concerns about the privacy and security of their data. When asked what additional features they expect from the CTA, some mentioned the need to understand the benefits of using it actively. When asked about their most common activities on their mobile devices, 76 participants stated that they are used for communication, 66 stated social networking, 60 stated entertainment purposes, and 40 indicated uses related to health and well-being.

### Assessment of the Scale

The HCTS under investigation includes five constructs: risk perception, competency, benevolence, reciprocity, and trust [20,22,26] (see Figure 1). Following the recommendation of Hair et al [29], the minimum sample size needed to effectively perform a PLS-SEM for our study was calculated to be 40 (ie, 10 times the maximum number of arrowheads pointing at a latent variable in a PLS path model). This method was selected because measuring trust in technology is complex, including four constructs and model relationships in this case. The measures used in the study were adapted from Gulati et al [20]. Their work models trust in technology with different studies, including trust in Siri using design fiction (future scenarios), the Estonian electronic voting service, and trust in human-robot interactions [24]. Gulati et al [20] measured risk perception using the concept of willingness and motivation developed through two independent studies [6,24]. This study added two additional items created through Schoorman et al’s [21] conceptualizations of trust. Gulati et al [20] measured competency and reciprocity based on the methodology of Mcknight et al [30], and measured benevolence based on adaptation of the prior work of Harwood and Garry [31] and McKnight et al [30]. The survey used a 7-point Likert scale to collect data, where 1 indicates strongly disagree and 7 indicates strongly agree. All of the items were positively worded except for the risk perception scale, which was adapted as a negatively worded statement and reversed before analyzing the data. The HCTS measures are summarized in Textbox 2.

**Human-Computer Trust Scale measures.**

**Risk perception**

RP1: I believe that there could be negative consequences from using HOIA

RP2: I feel I must be cautious when using HOIA

RP3: It is risky to interact with HOIA

RP4: I feel unsafe to interact with HOIA

RP5: I feel vulnerable when I interact with HOIA

**Competency**

COM1: I believe HOIA is competent and effective in identifying if I have been in close contact with a COVID-19–positive person

COM2: I believe HOIA has all the functionalities I would expect from a COVID-19 contact-tracing system

COM3: I believe that HOIA performs its role as a warning for close contacts with a COVID-19–positive person

**Reciprocity**

REC1: When I share something with HOIA, I expect to get back a knowledgeable and meaningful response

REC2: When sharing something with HOIA I believe that I will get an answer

**Benevolence**

BEN1: I believe HOIA acts in my best interest

BEN2: I believe that HOIA would do its best to help me if I need help

BEN3: I believe that HOIA is interested in understanding my needs and preferences

**General trust**

GT1: When I use HOIA, I feel I can depend on it completely

GT2: I can always rely on HOIA for guidance and assistance

GT3: I can trust the information presented to me by HOIA

### Data Analysis

**Figure 2:**
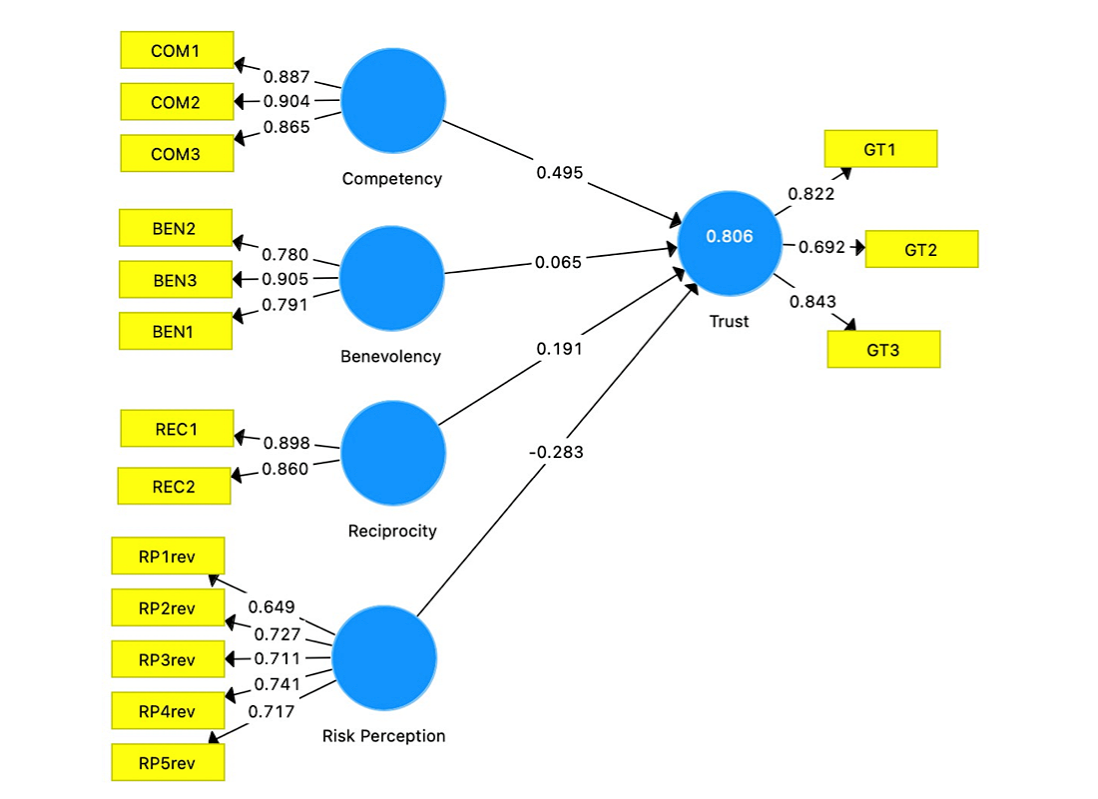
Final theoretical model loadings. BEN: benevolence; COM: competence; GT: general trust; REC: reciprocity; rev: reverse; RP: risk perception.

We analyzed a total of 78 answers. All scales for analyzing data in our study were positively worded, except perceived risk, which was negatively worded. The first steps in the analyses involved assessing the reliability and validity of the HCTS to measure trust in HOIA. In this phase, we calculated if the items have good measurements of the latent construct [29,32]. We discarded risk perception item 6 and competency item 4 because the loadings were below 0.5, and kept all loadings above their respective thresholds (>0.5). Table 1 and Figure 2 demonstrate all items used in the analysis and their loadings.

We further verified if the average variance extracted (AVE) was higher than 0.5; as shown in Table 1, all AVE values were >0.5, demonstrating that the items have good convergent reliability [12,32]. Similarly, the composite reliability of all indicators was above >0.7, showing adequate internal consistency. The Dillon-Goldstein ρ statistic, according to Hair et al [29], is similar to Cronbach α but allows the indicator variables to have varying outer loadings, and should be higher than 0.7 (or >0.6 in exploratory research). These values were above 0.7 for all items (Table 1), further demonstrating that the model is acceptable and has satisfactory internal consistency.

The discriminant validity and cross-loading values obtained using the Fornell-Lacker criterion (Table 2) indicated that the validity of each construct is higher for itself than for each corresponding construct [32].

**Table 1:** Loadings, reliability, and validity of the measurement model.

Items			Loadings (>0.5)	AVE^a^ (>0.5)			CR^b^ (>0.7)		Dillon-Goldstein ρ (>0.7)	
**Benevolence**					0.684			0.866		0.787
	BEN1	0.780								
	BEN2	0.905								
	BEN3	0.791								
**Competence**					0.784			0.916		0.864
	COM1	0.887								
	COM2	0.904								
	COM3	0.865								
**Reciprocity**					0.773			0.872		0.719
	REC1	0.898								
	REC2	0.860								
**Risk perception**					0.504			0.835		0.810
	RP1	0.649								
	RP2	0.727								
	RP3	0.711								
	RP4	0.741								
	RP5	0.717								
**Trust**					0.622			0.830		0.717
	GT1	0.822								
	GT2	0.692								
	GT3	0.843								

^a^AVE: average variance extracted.

^b^CR: composite reliability.

**Table 2:** Discriminant validity and cross-loading values (diagonal, italics) of the measurement items based on the Fornell-Lacker criterion.

Item	Benevolence	Competence	Reciprocity	Risk perception	Trust
Benevolence	*0.827*	0.747	0.620	–0.625	0.730
Competence	0.747	*0.885*	0.700	–0.585	0.843
Reciprocity	0.620	0.700	*0.879*	–0.526	0.727
Risk perception	–0.625	–0.585	–0.526	*0.710*	–0.714
Trust	0.730	0.843	0.727	–0.714	*0.789*

### Trust Toward HOIA

In addition, we assessed the coefficient of determination (*R^2^*) values, which represent the combined effect of exogenous latent variables on the endogenous latent variable, and is interpreted in the same way as in a conventional regression analysis procedure [29]. In this study, the *R^2^* value was 0.806 and the adjusted *R^2^* was 0.795. According to Hair et al [29], *R^2^* values of 0.75, 0.50, or 0.25 are considered substantial, moderate, or weak, respectively. In line with this interpretation, both the *R^2^* and adjusted *R^2^* values of this study indicate a substantial effect. Thus, approximately 83% of the changes in technology trust can be explained by the statistically significant exogenous variables in the HCTS. Accordingly, we conclude that the statistically significant attributes significantly predict user trust in COVID-19 CTAs, namely HOIA. Keeping in mind all of the empirical values obtained thus far, it is safe to say that our model passes the criteria for both measurement and structural model evaluation, and the final scale exhibits good validity, reliability, and predictive power.

## Discussion

### Principal Findings

To contribute toward our central research question (can the HCTS be used to assess an individual’s perception of trust in health technologies?), we empirically assessed the suitability of the HCTS to assess an individual’s perception of trust in health technologies, with the broader goal of understanding which attributes of the HCTS hold true in health technologies. As shown in Table 3, all but one of our four hypotheses were supported, based on statistically significant effects.

**Table 3:** Significance testing of structural model path coefficients.

Hypothesis	Path coefficient (SD)	*t* value	*P* value	97.5% CI	Significance (*P*<.0.5)
Benevolence mediates trust	0.062 (0.097)	0.674	.50	0.251	No
Competency mediates trust	0.495 (0.099)	5.022	<.001	0.690	Yes
Reciprocity mediates trust	0.195 (0.084)	2.285	.02	0.355	Yes
Risk perception mediates trust	–0.287 (0.056)	5.106	<.001	–0.197	Yes

For instance, H1 (risk perception mediates trust), H2 (competency mediates trust), and H4 (reciprocity mediates trust) were statistically significant, which is in line with the work of Gulati et al [20]. However, we also found that H3 (benevolence mediates trust) was nonsignificant (*P*=.52). To understand these results, it is important to consider how these constructs were operationalized. H1 and H2 were operationalized based on Gulati et al’s [20] and Schoorman et al’s [21] conceptualizations of trust, whereas H3 and H4 were operationalized based on Gulati et al [20].

### Limitations

Our study is not without its limitations, which can guide future research. First, culture influences trust. Second, the proposed scale (HCTS) demonstrated that trust is a dynamic construct that evolves in context and is culturally dependent. Third, the additional suggested items based on Schoorman et al’s [21] conceptualizations need further reassessment, as the results are more in line with those of Gulati et al [20], but also indicate no significant correlation between the Estonian citizens’ perception of HOIA as a benevolent trait.

### Conclusion

In conclusion, the results of this study indicate that the degree of trust toward the Estonian CTA (HOIA) is significantly correlated with the extent to which users perceive the system as competent, reciprocal, and risky. This study used PLS-SEM to identify statistically significant factors for assessing individuals’ perception of trust in human-technology interactions for health. This work contributes toward establishing a final version of the scale derived from the HCTS consisting of 13 items that can be used to measure user trust levels, including competence, reciprocity, and perceived risk. Moreover, these results should not only be limited to HOIA but can also be implemented to measure trust in other CTAs.

## Abbreviations

AI: artificial intelligence
AVE: average variance extracted
CTA: contact-tracing app
HCTS: Human Computer Trust Scale
HOIA: Estonian contact-tracing app for COVID-19
PLS-SEM: partial least-squares structural equation modeling
